# Embryonic exposure to environmental factors drives transmitter switching in the neonatal mouse cortex causing autistic-like adult behavior

**DOI:** 10.1073/pnas.2406928121

**Published:** 2024-08-23

**Authors:** Swetha K. Godavarthi, Hui-quan Li, Marta Pratelli, Nicholas C. Spitzer

**Affiliations:** ^a^Neurobiology Department, University of California San Diego, La Jolla, CA 92093; ^b^Kavli Institute for Brain & Mind, University of California San Diego, La Jolla, CA 92093

**Keywords:** autism spectrum disorders, valproic acid, poly inosine:cytosine, environmental models of autism, neurotransmitter switch

## Abstract

Autism spectrum disorders (ASD) can be caused by environmental factors. These factors act early in the development of the nervous system and induce stereotyped repetitive behaviors and diminished social interactions, among other outcomes. Little is known about how these behaviors are produced. In pregnant women, delivery of valproic acid (VPA) (to control seizure activity or stabilize mood) or immune activation by a virus increases the incidence of ASD in offspring. We found that either VPA or Poly Inosine:Cytosine (which mimics a viral infection), administered at mouse embryonic day 12.5, induced a neurotransmitter switch from GABA to glutamate in PV- and CCK-expressing interneurons in the medial prefrontal cortex by postnatal day 10. The switch was present for only a brief period during early postnatal development, observed in male and female mice at postnatal day 21 and reversed in both males and females by postnatal day 30. At postnatal day 90, male mice exhibited stereotyped repetitive behaviors and diminished social interaction while female mice exhibited only stereotyped repetitive behavior. Transfecting GAD1 in PV- and CCK-expressing interneurons at postnatal day 10, to reintroduce GABA expression, overrode the switch and prevented expression of autistic-like behavior. These findings point to an important role of neurotransmitter switching in mediating the environmental causes of autism.

## Significance

What is the mechanism by which different environmental agents induce a brief GABA-to-glutamate neurotransmitter switch in the neonatal brain, leading to autistic-like behavior in adult mice? GABA and glutamate are typically inhibitory and excitatory neurotransmitters, respectively. Switching their normal expression early in the assembly of the nervous system is expected to alter action potential frequencies that regulate activity-dependent components of neuronal development, including brain wiring. GABA is also a trophic factor and its disappearance is likely to affect further aspects of development. Several genetic mutations that cause autism spectrum disorder exhibit similar early imbalance in expression of these excitatory and inhibitory transmitters, suggesting that transmitter switching may be common to both environmental and genetic forms of this affliction.

Autism spectrum disorders (ASD) are characterized by varying degrees of enhanced stereotypic behaviors, deficits in social interaction, and defects in communication (1). Several susceptibility genes along with epigenetic and early environmental factors have been identified to play a key role (2). Despite this heterogeneous etiology, cortical imbalance in the expression and activity of inhibitory and excitatory neurotransmitters is a convergent phenotype that has been found in different forms of autistic behavior (3–8). For example, expression of GABA and glutamate is imbalanced in patients and animal models of ASD (9–11).

The transmitter expressed by a neuron is not fixed, but is capable of switching in response to changes in electrical activity both during development and in adults (12–17). Neurotransmitter switching frequently involves downregulation of one transmitter and upregulation of another, with matching changes in postsynaptic receptors (16, 18). Significantly, neurotransmitter switching often leads to changes in behavior (19–22), perhaps because the sign of the synapses by switching neurons changes from excitatory to inhibitory or vice versa (12, 21). The role for transmitter switching in disorders of the nervous system is now beginning to be understood (16, 23–27).

We hypothesized that transmitter switching is responsible for the emergence of chemical imbalance that causes the stereotypic behaviors and social avoidance observed in ASD. We tested this hypothesis using two established environmental models of autism. Pregnant mice were treated at embryonic day 12.5 with either valproic acid (VPA, an anticonvulsant) or Poly Inosine:Cytosine (Poly I:C, to induce maternal immune activation) (28, 29). We investigated transmitter switching in the medial prefrontal cortex (mPFC), a region known to be affected in ASD (30, 31).

We identified a transient GABA-to-glutamate switch in response to embryonic exposure to VPA or Poly I:C in a subset of interneurons in the mPFC of neonatal mouse pups. Adult mice demonstrated enhanced stereotypic behaviors and deficits in social interaction in a sex-dependent manner. Reintroducing GAD1 into the same neurons postnatally with viral tools to override the switch rescued normal adult behavior in male and female mice. These results are consistent with evidence that alteration in electrical signaling in the nervous system during the early stages of its construction can have negative consequences for the function of the mature brain (32–36).

## Results

### Embryonic Exposure to VPA or Poly I:C Alters Neurotransmitter Identity in Neonatal Mice.

**Fig. 1.**
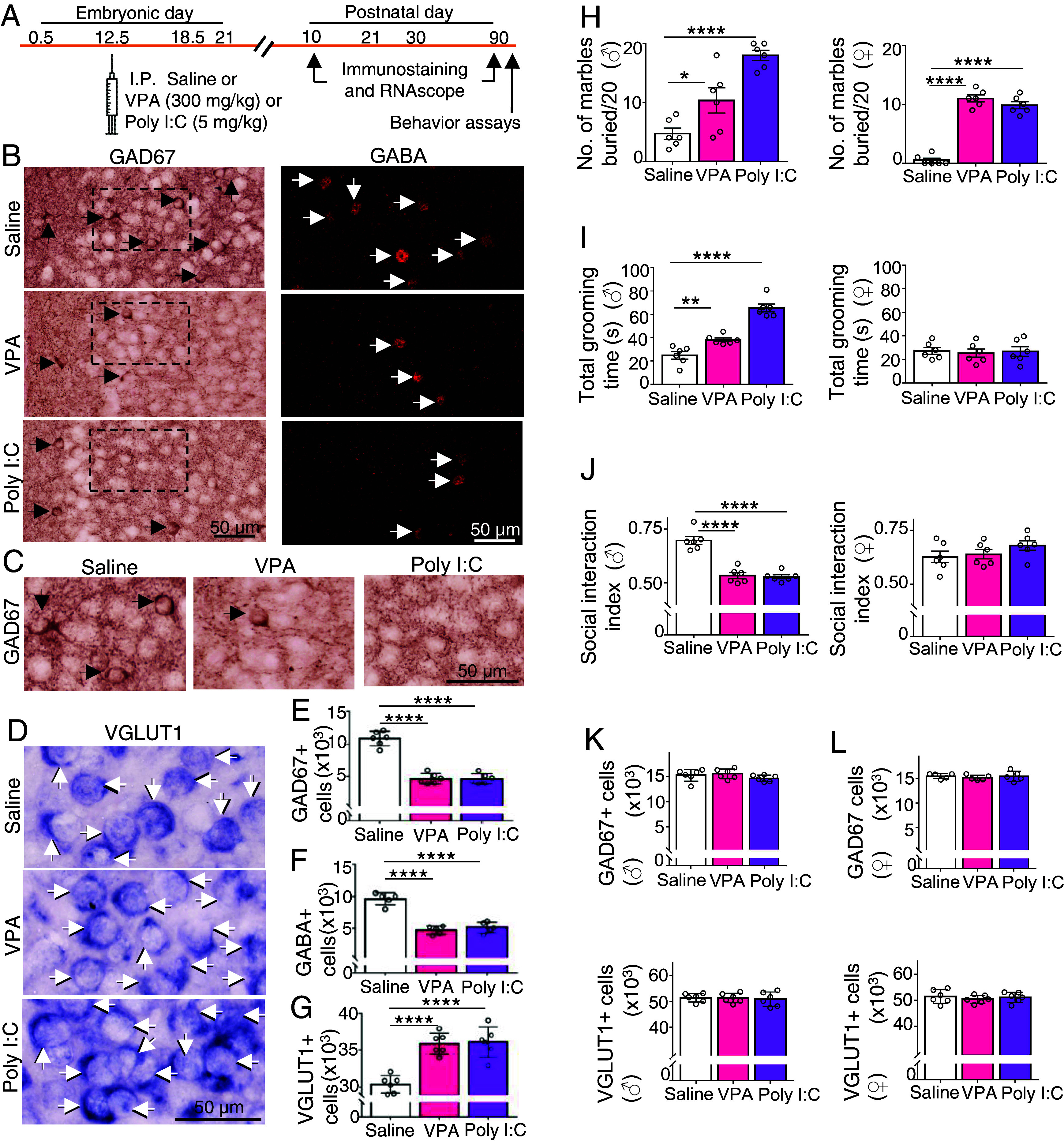
Prenatal exposure to VPA or Poly I:C changes the number of GABAergic and glutamatergic cells in the mPFC of P10 postnatal mice and changes the behavioral phenotype of P90 adults although the differences in transmitter identity are no longer present. (*A*) Experimental protocol. Following a single intraperitoneal (IP) dose of saline, VPA, or Poly I:C administered to pregnant dams at embryonic day (E) 12.5, mice were perfused at P10 or P90 for immunostaining and RNAscope. (*B*–*D*) Postnatal day (P) 10 mice from saline, VPA, and Poly I:C groups were processed for immunostaining for GAD67 (*B*, *Left*), GABA (*B*, *Right*), or RNAscope with VGLUT1 (*D*). Arrows indicate positive cells. (*C*) Higher magnification of GAD67 stain in dashed boxes in *B*. (*E*–*G*) Stereological counts of GAD67+ cells GABA+ cells and VGLUT1+ cells in P10 mice. (*H*) Number of marbles buried during a 10-min test period is higher in adult (P90) VPA- and Poly I:C-treated mice compared to saline controls for both males (♂, *Left* graph) and females (♀, *Right* graph). (*I*) *Left*, adult VPA- and Poly I:C-treated male mice spend more time grooming during a 10-min test period compared to controls. *Right*, no difference in grooming time across female groups of mice. (*J*) *Left*, control male mice show preference for social stimulus (conspecific) over novel object (social interaction index) in a 10-min three-chamber social interaction test. No preference for social stimulus for VPA- and Poly I:C-treated male mice. *Right*, females treated with VPA or Poly I:C exhibit preference for social stimulus, like controls. (*K* and *L*) Stereological counts of GAD67+ cells and VGLUT1+ cells in P90 males (*K*) and females (*L*). n ≥ 5 mice (*Materials and Methods*). *P < 0.05; **P < 0.01; ***P < 0.001; ****P < 0.0001, one-way ANOVA followed by the multiple comparison test (see Dataset S1 for details). Error bars: SD.

Management of seizures in pregnant women with VPA or maternal viral infection during pregnancy has been associated with increased incidence of ASD and schizophrenia in their offspring (37–39). Because ASD is a developmental disorder (40), we used mouse models of these environmental influences to investigate the underlying brain plasticity in newborn pups. We injected pregnant mice at E12.5 with saline, VPA, or Poly I:C (Fig. 1*A*). The timing of this treatment elicits autistic-like behaviors in adult male and female mice (28, 29, 41). Because the GABA switch from depolarizing to hyperpolarizing occurs between P2 and P9 in mice (42, 43) and reliable PV expression occurs in mice at P10 (44), we began our investigation of changes in transmitter identity at P10. We started at this early stage in order to reveal the primary or near-primary cause of ASD-like behavior induced by VPA and by Poly I:C and avoid study of secondary, tertiary, or later consequences of the primary change. We immunostained brain sections for GAD67, GABA, and VGLUT1 and focused on the mPFC where signaling appears disrupted in autistic individuals and in mouse models of autism (45).

We observed a decrease in the number of neurons expressing GAD67 and GABA and an equal increase in the number of neurons expressing VGLUT1 in the mPFC of P10 mice born to mothers that had received injections of VPA or Poly I:C, relative to control mice from pregnant mothers that had received saline injections. Stereological scoring confirmed this observation, indicating a loss of ~5,000 neurons stained for GAD67 and GABA and a gain of ~5,000 neurons stained for VGLUT1 in response to either drug (Fig. 1 *B*–*E* and Dataset S1).

We found that changes in the populations of neural progenitor cells could not account for the observed changes in numbers of GABAergic and glutamatergic neurons. We stained the telencephalon of pups of saline- or drug-treated dams at embryonic (E) 13.5 mouse for Tbr1 and at E18.5 for Dlx2. These transcription factors become expressed by E12.5 and identify precursors of glutamatergic and GABAergic neurons, respectively (46, 47). Tbr1 is expressed in the cortical plate by E13.5 but telencephalic neurons express Dlx2 from E16.5 onward. No differences were observed in the numbers of Tbr1-stained and Dlx2-stained neurons between saline- and drug-treated embryos (*SI Appendix*, Fig. S1 *A*–*D* and Dataset S2). At P10 there was no change in the aggregate population of GABAergic and glutamatergic neurons (Fig. 1 *B*–*E* and Dataset S1) nor in the NeuN+ population (*SI Appendix*, Fig. S1 *E* and *F* and Dataset S2) and evidence for apoptosis was absent (*SI Appendix*, Fig. S1 *G* and *H* and Dataset S2). However, at P10 there was no change in the number of neurons expressing GAD67 in the lateral prefrontal cortex or the number of neurons expressing nitric oxide synthase 1 (NOS1) in the mPFC (*SI Appendix*, Fig. S2 *A*–*D* and Dataset S2). These results are consistent with a change in transmitter identity specific to a subset of GABAergic neurons in the neonatal mPFC.

### Autistic-Like Behavior Develops and Persists to at Least P90.

We evaluated autistic-like behavior using two tests for stereotypical behavior and one test of social interaction. Individuals with ASD typically exhibit increased repetitive behaviors and decreased interest in interaction with conspecifics. As expected (28, 29, 48), relative to saline controls, adult mice at P90, exposed embryonically to VPA or Poly I:C, exhibited increased stereotypic behavior. In the marble-burying test (49), the mouse was placed in a cage for 10 min with 20 marbles arrayed on the cage bedding. Male control mice buried a mean of 5 marbles while VPA- and Poly I:C-treated male mice buried 10 and 18 marbles, respectively (Fig. 1*H* and Dataset S1). In the grooming test (50), grooming time in 10 min increased from 25 s in male controls to 38 s and 66 s in VPA- and Poly I:C-treated mice (Fig. 1*I* and Dataset S1). In the 10-min test of social interaction (50, 51), male control mice preferred interacting with a novel conspecific rather than a novel object (0.70 social interaction index), while VPA- and Poly I:C-treated mice displayed no preference (0.50 index for each treatment group; Fig. 1*J* and Dataset S1). Thus, adult male mice treated with VPA or Poly I:C exhibited abnormal behavior on all three tests. However, adult female mice were affected in only the marble-burying test (Fig. 1 *H*–*J* and Dataset S1). In the VPA and Poly I:C treatment groups females buried 11 and 10 marbles, respectively. Adult female saline control mice buried a single marble. This sex-specific difference in autistic-like behavior parallels observations of behavior in humans diagnosed with autism (52).

### The Change in Transmitter Identity Reverses Spontaneously by P30.

**Fig. 2.**
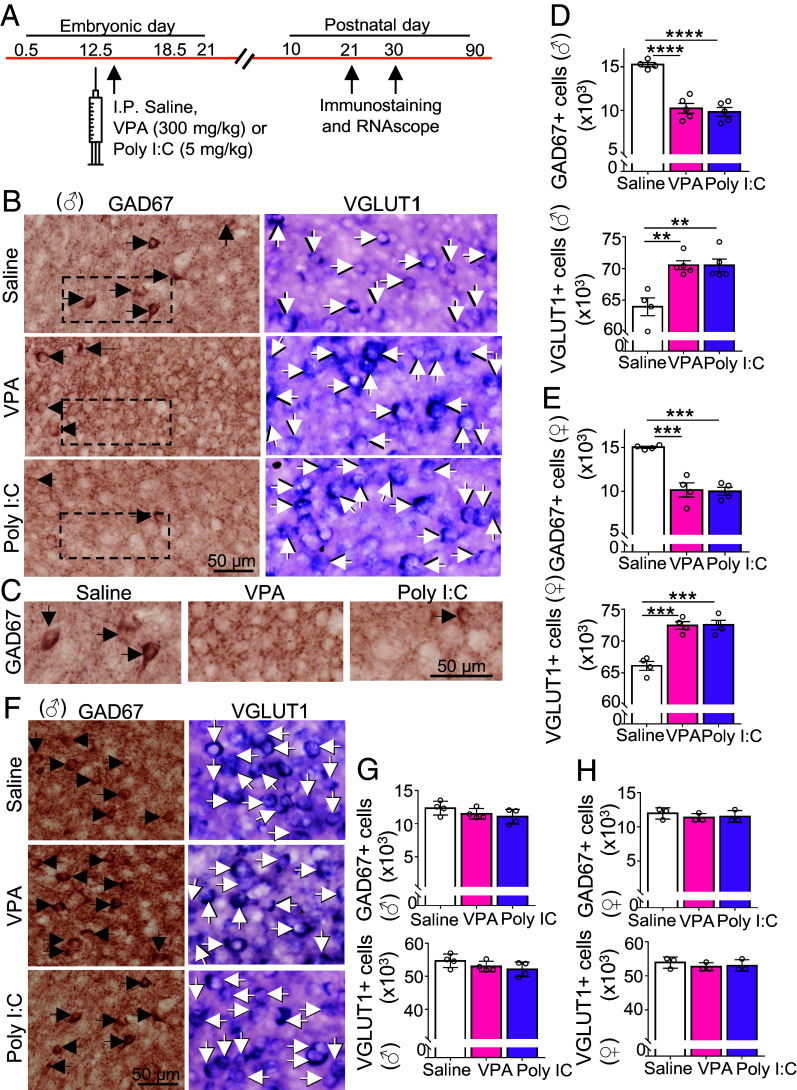
The change in transmitter identity is present in the mPFC at P21 but is not observed at P30. (*A*) Experimental protocol. Following a single IP dose of saline, VPA, or Poly I:C administered to pregnant dams at embryonic day (E) 12.5, mice were perfused at P21 or P30 for immunostaining and RNAscope. (*B*) Three-week postnatal (P21) males (♂) from saline, VPA-, and Poly I:C-treated groups were processed for immunostaining for GAD67 or RNAscope for VGLUT1. Arrows indicate positive cells. (*C*) Higher magnification of GAD67 in dashed boxes in *B*. (*D* and *E*) Stereological counts of GAD67+ and VGLUT1+ cells in P21 males (*D*) and females (*E*). (*F*) One-month-old (P30) males (♂) from saline, VPA-, and Poly I:C-treated groups were processed for immunostaining for GAD67 or RNAscope for VGLUT1. Arrows indicate positive cells. (*G* and *H*) Stereological counts of GAD67+ cells and VGLUT1+ cells in P30 males (*G*) and females (*H*). n ≥ 3 mice (*Materials and Methods*). ***P < 0.001; ****P < 0.0001, one-way ANOVA followed by the multiple comparison test (see Dataset S1 for details). Error bars: SD.

Notably, at P90 there was no difference in the mPFC, for either males or females, in the number of GABAergic neurons or in the number of glutamatergic neurons between saline control and drug-treated mice (Fig. 1 *K* and *L*, and Dataset S1). We found that the change in transmitter identity was present at P21 (Fig. 2 *A*–*E* and Dataset S1) but was not sustained at P30 (Fig. 2 *F*–*H* and Dataset S1). Both for males and females at this stage of development (P30), the number of neurons expressing GAD67 and the number of neurons expressing VGLUT1 were not different between saline, VPA-, and Poly I:C- treated mice. The return to the normal numbers of neurons stained for these transmitter markers may have resulted from metabolism and clearance of the drugs or from the closure of a critical period during which the drugs are effective. Staining for TUNEL, Ki67, and DCX at P25 did not detect evidence for apoptosis or for neurogenesis (*SI Appendix*, Fig. S3 and Dataset S2). These results suggest that there was a transient change from a GABAergic to glutamatergic phenotype in the mPFC, at least 11 d in duration (from P10 to P21), which reverses after P21.

### The Change in Transmitter Identity Occurs in Parvalbumin and Cholecystokinin Neurons.

**Fig. 3.**
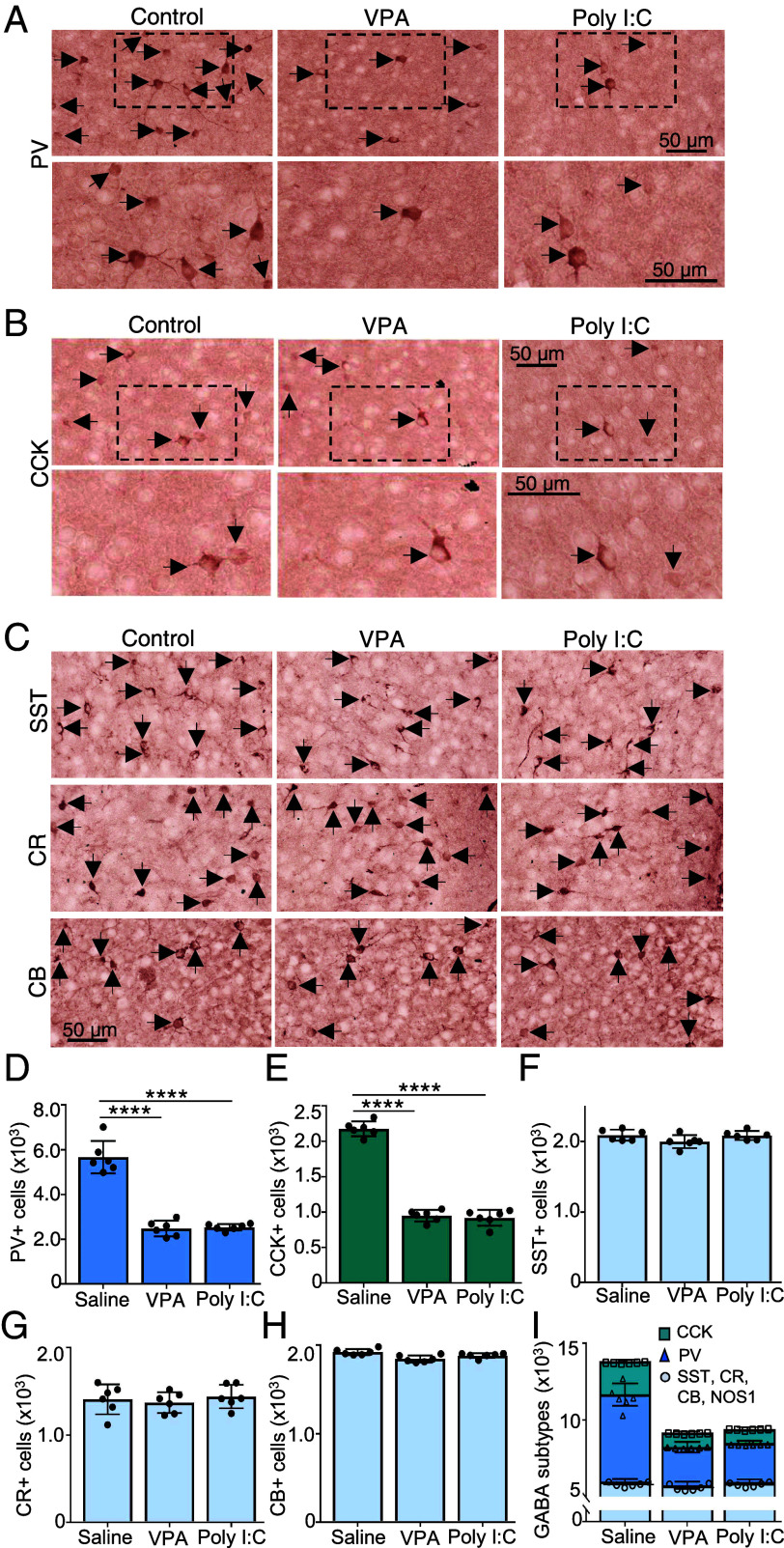
PV and CCK interneurons account for the decrease in the number of neurons expressing GAD67+ and GABA+ that matches the increase in the number of neurons expressing VGLUT1+ in the mPFC of VPA- and Poly I:C-treated mice. (*A*–*C*) Postnatal day (P) 10 C57Bl6 pups from saline, VPA-, and Poly I:C-treated dams were immunostained for PV (*A*), CCK (*B*), and somatostatin (SST), CR, and CB (*C*). Arrows indicate positive cells (see *SI Appendix*, Fig. S1 for NOS immunostaining). (*A* and *B*) *Lower* panels, higher magnifications of regions indicated by dashed boxes for PV (*A*) and CCK (*B*). (*D*–*I*) Stereological counting of PV (*D*), CCK (*E*), SST (*F*), CR (*G*), and CB (*H*) cells. (*I*) Combined counts of all the GABAergic cell subtypes analyzed—PV, CCK, and others (SST, CR, CB, and NOS1). n ≥ 6 mice for all. ****P < 0.0001 one-way ANOVA followed by the multiple comparison test (see Dataset S1 for details). Error bars: SD.

We next sought the identity of the neurons that gained expression of VGLUT1. GABAergic neurons in the mPFC coexpress a range of different calcium-binding proteins or neuropeptides (53). We followed maternal injection of VPA or Poly I:C with immunostaining of neurons at P12 for different calcium-binding proteins [calbindin (CB), calretinin (CR), parvalbumin (PV)] or peptide neuromodulators [somatostatin (SST), NOS, cholecystokinin (CCK)]. Stereological counts demonstrated a decrease at P12, in both males and females, in the number of PV and CCK neurons and no decrease in the number of CB, CR, SOM, and NOS1 neurons (Fig. 3 *A*–*H* and Dataset S1). Apoptosis was not detected (*SI Appendix*, Fig. S1*I* and Dataset S2) and PV neurons can survive after loss of PV (54, 55). The decrease in the number of PV and CCK neurons matched the decrease in the number of GABAergic neurons (Fig. 3*I* and Dataset S1) and the increase in the number of VGLUT1 neurons. These findings suggested that VGLUT1 appeared in neurons that formerly expressed PV and CCK. We observed a similar decrease in PV and CCK neurons in the absence of apoptosis at P21, suggesting continued expression of the glutamatergic phenotype in these neurons (*SI Appendix*, Fig. S4 and Dataset S2). Loss of PV basket cell interneurons occurs in subregions of the PFC in postmortem tissue from autistic subjects (56), along with loss of the GABA transporter, VGAT (57), with loss in one subregion correlated with the severity of stereotypic motor behavior (58). At P90 there was no difference in the number of PV neurons or CCK neurons between saline control and drug-treated mice (PV: saline 8,639 ± 412, VPA 8,264 ± 294 and Poly I:C 8,378 ± 474; CCK; saline 3,007 ± 108, VPA 2,904 ± 138 and Poly I:C 3,016 ± 82) (*SI Appendix*, Fig. S5 *A* and *B* and Dataset S2). The age at which PV and CCK expression reappears in VPA- and Poly I:C-treated mice remains to be determined.

### The Transmitter Switch Occurs in Single PV and CCK Neurons.

To determine first whether transmitter switching occurs in single GABAergic neurons, we treated pregnant VGATCre::ZsGreen mice with Poly I:C or saline at E12.5 and stained the mPFC for VLGUT1 and GAD1 at P10. The vesicular GABA transporter VGAT drove Zsgreen labeling in GAD1+ GABAergic neurons in the mPFC of saline-treated mice, and VGLUT1-labeled neurons did not express Zsgreen. However, VGAT drove Zsgreen labeling of cells that were VGLUT1+ but GAD1- in the mPFC of Poly I:C-treated mice (*SI Appendix*, Fig. S6 and Dataset S2). The Zsgreen label identified these neurons as previously GABAergic, while VGLUT1 suggested they were now glutamatergic. These results provide evidence for transmitter switching in single GABAergic neurons.

**Fig. 4.**
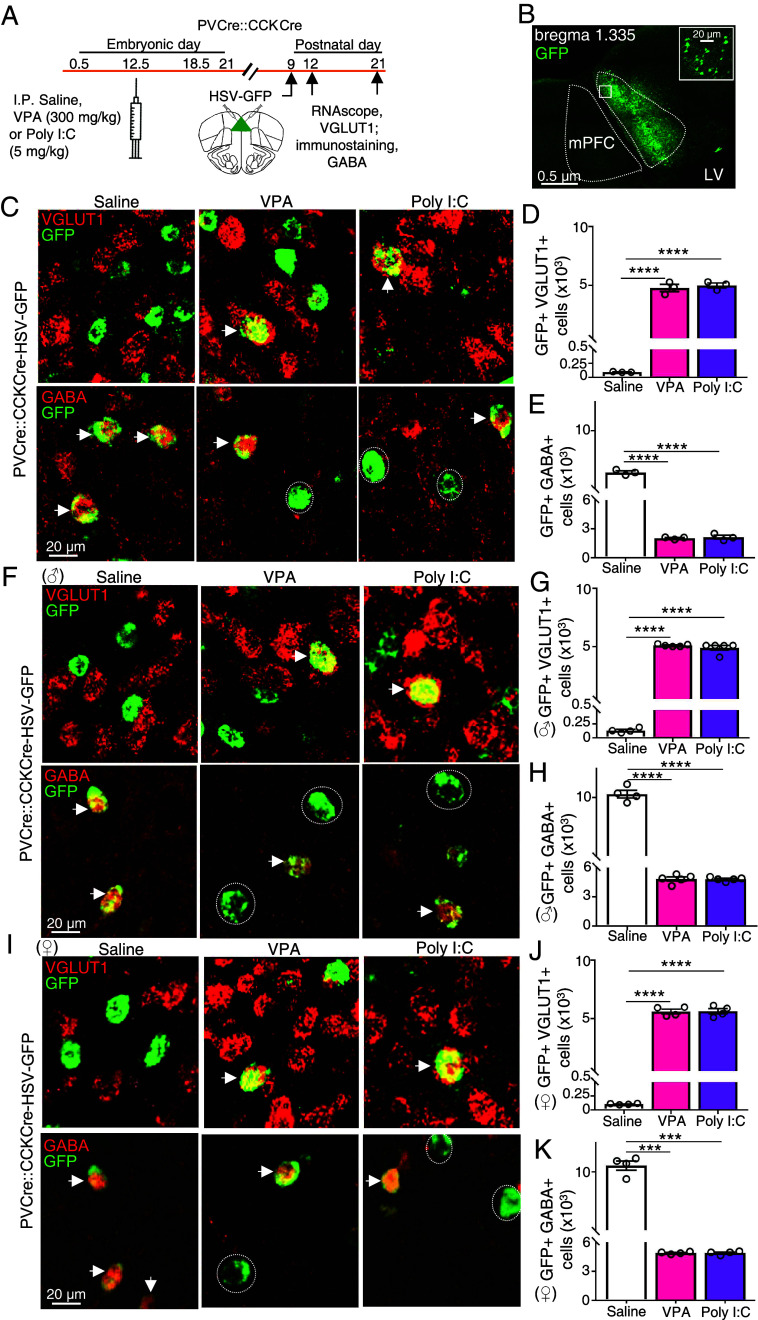
HSV-GFP transfection of the P9 mPFC of PVCre::CCKCre VPA- and Poly I:C-treated mice identifies a transmitter switch at the single-cell level in the mPFC at P12 and at P21. (*A*) Experimental protocol for treatment of PVCre::CCKCre transgenic mice. Following a single IP injection of saline, VPA, or Poly I:C at embryonic day (E) 12.5, mice were injected in the mPFC with Cre-dependent HSV-GFP at P9 and processed at P12 and P21 for RNAscope for VGLUT1 and immunostaining for GABA. (*B*) GFP expression in the P12 mPFC (dotted lines) following unilateral HSV-GFP injection in PVCre::CCKCre mice. The *Inset* shows higher magnification of the boxed area. LV, lateral ventricle. (*C*) GFP expression in the P12 mPFC following bilateral HSV-GFP injection, stained for VGLUT1 (*Top* panels) or for GABA (*Bottom* panels). Arrows indicate GFP+VGLUT1+ or GFP+GABA+ cells. Dotted circles indicate GFP+GABA- cells. (*D* and *E*) Counts of GFP+VGLUT1+ cells (*D*) and counts of GFP+GABA+ cells (*E*) per mouse. n = 3 mice. ****P < 0.0001, unpaired two-tailed *t*-test. (*F*) P21 PVCre::CCKCre male mice injected with HSV-GFP from saline, VPA-, and Poly I:C-treated groups were processed for RNAscope for VGLUT1 (*Top* panels) or immunostained for GABA (*Bottom* panels). Arrows indicate GFP+VGLUT1+ or GFP+GABA+ cells. Dotted circles indicate GFP+GABA− cells. (*G* and *H*) Counts of GFP+VGLUT1+ cells (*G*) GFP+GABA+ cells (*H*) per mouse. n ≥ 3 mice. (*I*) P21 PVCre::CCKCre female mice injected with HSV-GFP from control, VPA- and Poly I:C-treated groups were processed for RNAscope for VGLUT1 (*Top* panels) or immunostained for GABA (*Bottom* panels). Arrows indicate GFP+VGLUT1+ or GFP+GABA+ cells. Dotted circles indicate GFP+GABA- cells. (*J* and *K*) Counts of GFP+VGLUT1+ cells (*J*) GFP+GABA+ cells (*K*) per mouse. n ≥ 3 mice (*Materials and Methods*). ***P < 0.001, ****P < 0.0001, one-way ANOVA followed by the multiple comparison test (see Dataset S1 for details). Error bars: SD.

To learn next whether transmitter switching occurs in single GABAergic neurons that coexpress either PV or CCK, we next generated a PV-Cre::CCK-Cre mouse line in order to label PV and CCK neurons with Cre-dependent GFP and examine VGLUT1 expression in GFP+ neurons. The specificities of PV-Cre and CCK-Cre lines were determined in male mice and found to be 97 ± 3% and 90 ± 4%, respectively (*SI Appendix*, Fig. S5 *C*, *D*, *F*, *G*, and *I*–*K*) (*Materials and Methods*). We then immunostained Cre, PV, and CCK in sections of P10 PV-Cre and CCK-Cre mice treated with VPA or Poly I:C, to determine whether neurons that have lost PV or CCK continue to express Cre. We also immunostained Cre and probed for PV and CCK, to determine whether neurons that have lost detectable PV or CCK transcripts continue to express Cre. These experiments demonstrated that, compared to saline controls, more cells are Cre+ but PV- and CCK- in VPA- and Poly I:C-treated mice (*SI Appendix*, Figs. S7 and S8, and Dataset S2). Herpes simplex virus (HSV) is an attractive vector with which to introduce GFP, because it expresses rapidly, within a few days (59), as required for a transmitter switch that persists for only a brief period. The efficiency of expression of HSV-DIO-GFP in the PV-Cre::CCK-Cre line was found to be 92% (*SI Appendix*, Fig. S5 *D*, *G*, and *L*) (*Materials and Methods*). The decrease in GABA and increase in VGLUT1 were recapitulated in PV-Cre::CCK-Cre transgenic mice (*SI Appendix*, Fig. S9 and Dataset S2). Stereotaxic injection of P9 PV-Cre::CCK-Cre VPA- and Poly I:C-treated embryos with Cre-dependent HSV-GFP revealed a population of neurons in the neonatal mPFC at P12 and P21 that expressed GFP and VGLUT but not GABA (Fig. 4 *A*–*K* and Dataset S1). These experiments demonstrate in male and female mice that a subset of PV-Cre::CCK-Cre GABAergic neurons express VGLUT1 in response to maternal Poly I:C or VPA treatment, with concomitant loss of GABA expression, providing evidence that neurotransmitter switching occurs in single PV neurons and CCK neurons.

### Overriding the Transmitter Switch in Neonates Prevents the Development of Autistic-Like Behavior in Adult Mice.

**Fig. 5.**
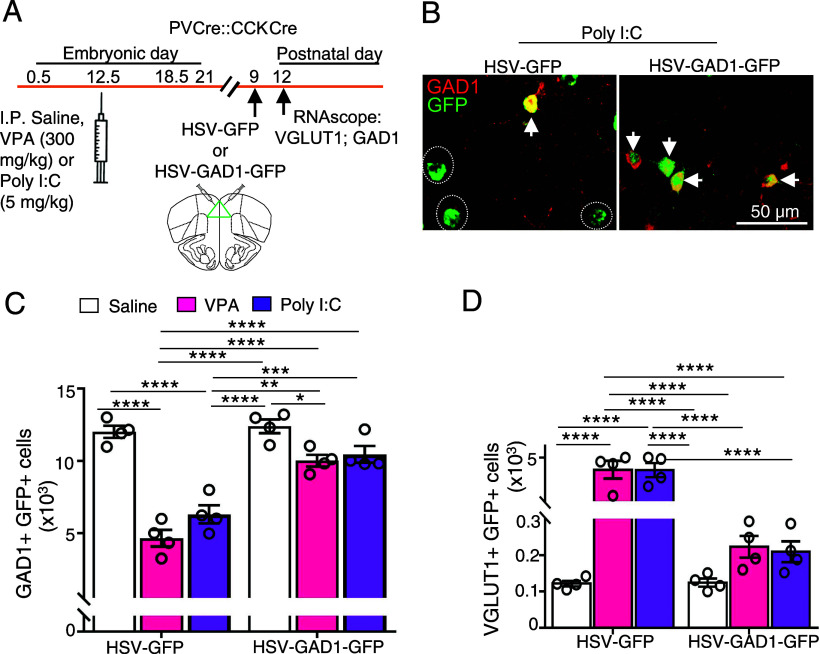
Expression of GAD1 in PV and CCK neurons rescues loss of GAD1 and gain of VGLUT1. (*A*) Experimental protocol. Following a single IP dose of saline, VPA, or Poly I:C in pregnant dams at E 12.5, PVCre-CCKCre transgenic mice were injected bilaterally with Cre-dependent HSV-GFP or HSV-GAD1-GFP at P9 and perfused at P12 for RNAscope for GAD1 and VGLUT1. (*B*) Images of GAD1+ neurons in Poly I:C treated PVCre-CCKCre mice injected with HSV-GFP or HSV-GAD1-GFP. Arrows indicate GFP+GAD1+ cells. Dotted circles indicate GFP+GAD1− cells. (*C* and *D*) Stereological counts of neurons of P12 PVCre-CCKCre mice injected with HSV-GFP or HSV-GAD1-GFP from saline, VPA-, and Poly I:C-treated groups and processed for RNAscope for GAD1 (*C*) or VGLUT1 (*D*). n ≥ 3 (*Materials and Methods*). *P < 0.05, **P < 0.01, **P < 0.001, ****P < 0.0001, two-way ANOVA followed by multiple comparison (see Dataset S1 for details).

**Fig. 6.**
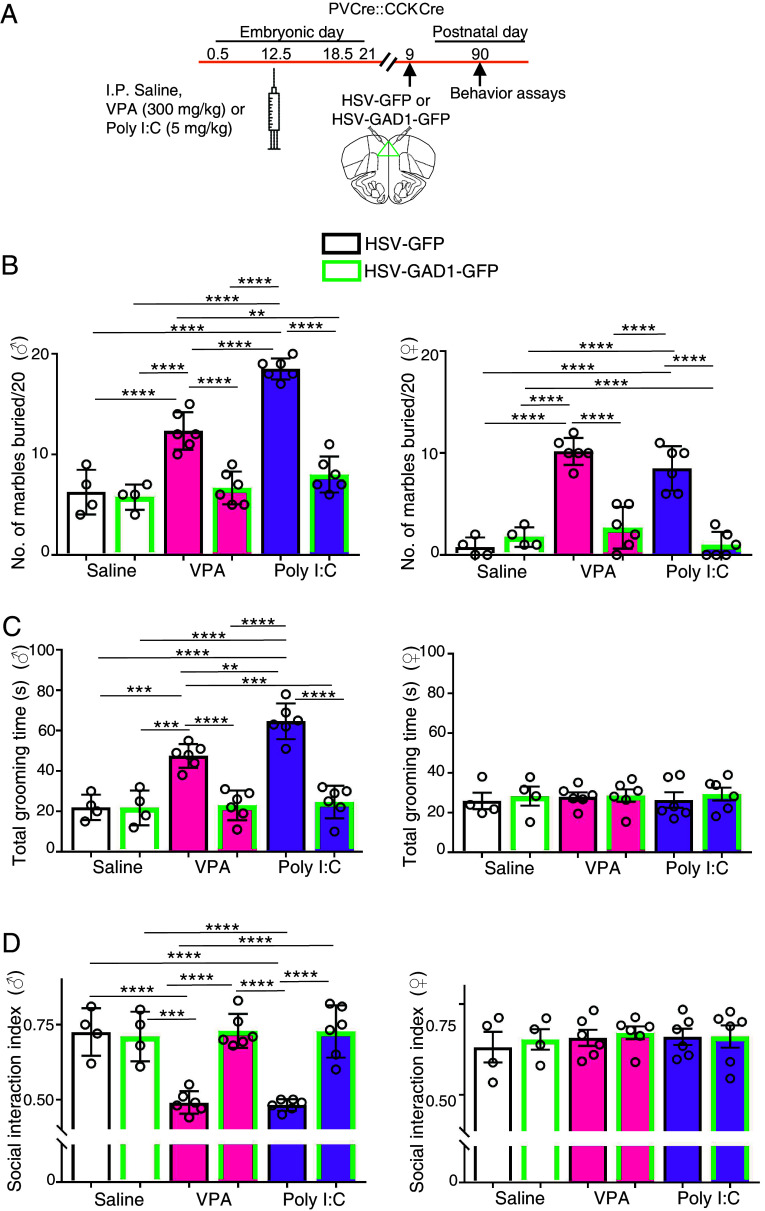
Expression of GAD1 in the P9 neonatal mPFC of PVCre::CCKCre VPA- and Poly I:C-treated mice rescues behavioral phenotypes of P90 adults. (*A*) Experimental protocol. Following a single IP dose of saline, VPA, or Poly I:C administered to pregnant dams at embryonic day (E) 12.5, PVCre-CCKCre transgenic mice were injected bilaterally with Cre-dependent HSV-GFP or HSV-GAD1-GFP at P9 and behavior was analyzed at P90. (*B*) Total number of marbles buried at the end of a 20 min test period by GAD1-GFP-expressing VPA- and Poly I:C-treated animals (green border) is similar to control groups (saline-treated mice expressing either HSV-GFP or HSV-GFP-GAD1) for both males (♂, *Left* graph) and females (♀, *Right* graph). (*C*) *Left*, adult GAD1-GFP-expressing VPA- and Poly I:C-treated male mice and control mice spend less time grooming compared to GFP-expressing VPA- and Poly I:C-treated mice. *Right*, no difference in grooming time across female groups of mice. (*D*) *Left*, control male mice show preference for social stimulus (conspecific) over inanimate object (social interaction index) in a three-chamber social interaction test. Preference for social stimulus is at chance level for VPA- and Poly I:C-treated male mice and is similar to saline controls in GAD-GFP-expressing VPA- and Poly I:C-treated mice. *Right*, female mice treated with VPA or Poly I:C continue to exhibit preference for social stimulus similar to controls. n ≥ 4 mice (*Materials and Methods*). **P < 0.01; ***P < 0.001; ****P < 0.0001, two-way ANOVA (multiple comparison) (see Dataset S1 for details). Error bars: SD.

To test the role of the transmitter switch in generating autistic behaviors, we restored GAD67 specifically in PV and CCK neurons in the mPFC. Previous work demonstrated that overriding one of the two transmitter changes in a switch can prevent the change in behavior associated with the switch (21). We stereotactically injected Cre-dependent HSV-GAD1-GFP in VPA- and Poly I:C-treated PV-Cre::CCK-Cre mice at P9 and scored the results at P12. GFP and GAD67 or GABA were colocalized in drug-treated mice. The expression of the GABAergic phenotype was coupled with reduced staining of VGLUT1 in GFP-labeled cells. In VPA- and Poly I:C-treated controls injected with HSV-GFP at P10, VGLUT1 staining colocalized with GFP and the GABA expression was absent at P12 (Fig. 5 *A*–*D* and Dataset S1). Restoring GAD67 expression in the mPFC of PV-Cre::CCK-Cre mice returned marble burying, grooming, and the social interaction index to control levels in adult male Poly I:C and VPA mice and returned marble burying to control levels in adult female VPA- and Poly I:C mice (Fig. 6 *A*–*D* and Dataset S1). These results provide evidence for the contribution of an early, transient GABA-to-glutamate transmitter switch to changes in behavior that are characteristic of ASD.

## Discussion

Embryonic exposure to either VPA or Poly I:C causes a neurotransmitter switch, in which PV and CCK interneurons in the neonatal mPFC transiently lose their expression of GABA, GAD67, PV, and CCK and gain expression of VGLUT1. These mice exhibit autistic-like behaviors as adults. Expressing GAD1 in PV and CCK neurons in the neonatal mPFC prevents development of these behaviors. The switch from GABAergic to glutamatergic phenotype involves the loss of a neuropeptide (CCK) and a calcium buffer (PV). The different roles of the decrease in GABA, CCK, and PV and the increase in glutamate merit further investigation. Although each of these two drugs has other effects, both of them produce the same transmitter switch and the same changes in behavior, and the changes in behavior are prevented by overriding the transmitter switch produced by each drug. Whether stress plays a role in causing the transmitter switch, as reported in a different context (24), is unknown. The expression of GAD1 in neurons that already express it (nonswitching neurons) is unlikely to contribute to the restoration of normal behavior, since increased GABA levels cause a decrease in the level of GAD67 (60). It will be of interest to learn whether other environmental cues, or the same cues at different stages of development, affect transmitter identity in other populations of interneurons and contribute to other disorders.

Disruption of the PFC elicits behaviors that are symptomatic of ASD in human subjects (61). The mPFC undergoes extensive structural and functional development in the first 2 wk of development in the mouse (62) and exhibits a critical window for spontaneous synchronous activity between P0 and P15 that peaks between P9 and P10 (63). The change from inhibition to excitation of postsynaptic cells in response to VPA or Poly I:C will alter electrical activity that can regulate proliferation, migration, and differentiation in early neuronal development (64). These changes in inhibitory and excitatory synaptic transmission at early postnatal stages are likely to alter neuronal and glial differentiation and the assembly of brain circuitry, contributing to autistic behavior that appears later in development. Signaling by calcium and other second messengers that are abundant at early stages of development can have exceptional impact during critical periods of differentiation (65, 66). In addition to autism, there are numerous instances of environmental and genetic impacts on early development that alter behavior later in the young individual and in the adult.

The mechanisms by which VPA and Poly I:C drive the transmitter switch are presently unknown. However, early in neuronal development, the NKCC1 transporter imports chloride ions, elevates [Cl^-^]_i,_ and GABA is depolarizing. Both VPA (67) and Poly I:C (68, 69) increase the expression of NKCC1. Later in development, the KCC2 transporter exports chloride ions, lowers [Cl^−^]_i,_ and GABA is hyperpolarizing. Both Poly I:C (68) and VPA (70) decrease the expression of KCC2. Thus, both drugs may enhance the level and duration of neuronal depolarization by systemic release of GABA, potentially driving activity-dependent GABA-to-glutamate switching (25, 71). Bumetanide blocks NKCC1, preventing the import of chloride ions when administered early in development (32), and makes the action of GABA more hyperpolarizing. On this view, repeated bumetanide administration 1 d before delivery, to sustain suppression of increased activity, may prevent the activity-dependent transmitter switch. How the PV and CCK neurons are specifically affected by VPA and Poly I:C remains to be determined. Preferential sensitivity of their chloride cotransporters to these drugs, driving their electrical activity to a higher level than other interneurons, could be sufficient to elicit the transmitter switch.

The absence of a sex difference in transmitter switching in response to VPA or Poly I:C administration needs to be reconciled with the sex differences in behavior. Differences in the level of testosterone or the androgen-to-estrogen balance may regulate behavior (72) downstream of transmitter switching. VPA also induces sex-dependent differences in x-linked gene expression and in inflammation pathways (73). Poly I:C causes a decrease in anti-inflammatory factors in male mice and an increase in these factors in female mice (74). These findings are consistent with compensation by anti-inflammatory factors in female mice that renders some behaviors indistinguishable from normal. More research is needed to identify what may be multifactorial regulation of sex differences in autistic behaviors.

Mice with genetic mutations that cause ASD can exhibit developmentally expressed decreases in GABAergic markers or increases in glutamatergic markers in the cortex that parallel the GABA-to-glutamate transmitter switch described here. Mice null for *Cntnap2* have a reduced number of PV GABAergic neurons and GAD1 expression at P14 that is associated with defective migration and exhibit autistic behavior (75). Mice with haploinsufficiency of *Arid1b* have a reduced number of PV GABAergic neurons and GABA expression at P28 that is associated with reduced proliferation and enhanced apoptosis and show autistic behavior (76). Mice with haploinsufficiency of *Mdga2*, which is associated with reduced neuroligin-1–neurexin interaction and is normally expressed in cortex and in PV GABAergic neurons at P14, have increased density of excitatory synapses, synaptic surface AMPA receptor levels and early-LTP, and demonstrate autistic behavior (77). Recordings from Shank3B-mutant mice lacking the synaptic PDZ domain at P0 and P15 reveal increased frequency of excitatory mPSCs and decreased expression of PV in synaptic puncta (78). These mutations generate an early imbalance in excitatory and inhibitory transmitter expression that is similar to that produced by environmental factors, suggesting that transmitter switching may be common to both forms of autism.

## Materials and Methods

### Mice.

C57BL/6J (JAX#000664), PV-Cre (JAX#017320), CCK-IRES-Cre (JAX#012706), VGAT-IRES-Cre (JAX#028862), and B6.Cg-*Gt(ROSA)26Sor^tm6(CAG-ZsGreen1)Hze^*/J (ZsGreen, JAX#007906) male and female mice were obtained from Jackson Laboratories. Heterozygous VGAT-Cre animals were bred with homozygous ZsGreen mice to obtain VGAT-Cre+/−::ZsGreen+/− experimental mice. PV-Cre and CCK-Cre mice were maintained as homozygotes. All animal procedures were carried out in accordance with NIH guidelines and approved by the University of California, San Diego, Institutional Animal Care and Use Committee. Mice were maintained on a 12 h:12 h light:dark cycle (light on: 7:00 am to 7:00 pm) with ad libitum access to food (7912.15 Irradiated Teklad LM Mouse diet) and water. Temperature was maintained at ~23 °C with 40 to 60% humidity. Mice were preferentially housed 2 or 3 per cage with nesting material. After receiving surgery, mice were single-housed with nesting material. Age groups used for experiments on mouse offspring included postnatal day (P) P5, P9, P10, P12, P21, P30, and P90 mice. Identification of gender is difficult at P5 to P12, but male and female mice were investigated separately at P21, P30, and P90. We presume that males and females were included in the P5 to P12 populations. To generate environmental models of autism, pregnant females at E12.5 were injected intraperitoneally with either 5 mg/kg Poly IC or 300 mg/kg of VPA (or saline control). The pups born to these females were either anesthetized and perfused or used for behavioral assays at the indicated time points.

### Behavioral Assays.

For all behavioral experiments mice were habituated to the room and the experimenter for 1 wk before testing.

#### Marble burying assay.

The marble-burying assay is a simple but powerful test of the presence of repetitive behaviors in mice (49). It takes advantage of the proclivity of mice to dig in their natural environment and into standard cage beddings. We placed standard glass marbles on the surface of bedding in five rows of four marbles. The mouse was introduced at the corner of the cage and left undisturbed for 10 min. At the end of the test, the number of marbles buried by the animal was scored, providing a direct measure of repetitive burying behavior by the mouse.

#### Self-grooming assay.

Mice normally scratch and brush their hair with their forelimbs. However, when this self-grooming behavior is performed at a higher rate and for a longer duration it is considered repetitive behavior (50). A mouse was placed in a standard cage and its behavior was videotaped for 10 min. Self-grooming involves face wiping, rubbing of the head area including ears, cleaning of the entire body for a period of at least 10 s and scratching. Self-grooming time was scored for each video by two independent observers.

#### Social interaction assay.

We tested social interaction using the three-chambered social approach task (50, 51). This task employs a central chamber for introducing the test mouse, a second chamber containing the novel conspecific, and a third containing a novel object. Prior to the task, test and novel mice were weighed and paired for the task such that the conspecific novel mouse was matched to the test mouse with respect to gender, age, and weight (not heavier or lighter than 5% test mouse weight). The behavior of the test mouse was monitored for a period of 10 min.

### Histology.

Mice were deeply anesthetized with isoflurane vapor and perfused transcardially with phosphate-buffered saline (PBS) followed by 4% paraformaldehyde (PFA) in PBS. Brains were dissected and postfixed in 4% PFA overnight at 4 °C and transferred to 30% sucrose in PBS for 2 d at 4 °C. 30 μm thick coronal sections were obtained using a microtome (Leica SM2010R) and immunostained. For long-term preservation, sections were stored in cryoprotectant solution (30% glycerol, 30% ethylene glycol, 20% 0.2 M phosphate buffer) at −20 °C.

#### Immunohistochemistry.

For immunostaining, sections were permeabilized in 0.3% Triton X-100 in PBS and blocked in 24-well culture plates (blocking solution: 5% normal horse serum, 0.3% Triton X-100 in PBS) for 2 h at room temperature (22 to 24 °C). Primary and secondary antibodies were diluted in the blocking solution. Incubation with primary antibodies was performed overnight on a rotator at 4 °C. After washing in PBS (three times, 15 min each), secondary antibodies were added for 2 h at room temperature. For immunofluorescence, sections were mounted with Fluoromount-G (Southern Biotech) containing DRAQ-5 (Thermo Fisher, 62251, 1:1,000 dilution; when nuclear staining was needed) after washes in PBS (three times, 15 min each).

For DAB (3,3′-Diaminobenzidine) staining, sections were treated with 0.3% hydrogen peroxide for 30 min, washed in PBS (three times, 5 min each), incubated with Vectastain Elite ABC HRP mixture (Vector Laboratories, PK-6100) for 45 min, washed in PBS (three times, 15 min each), and signals were developed using the DAB Peroxidase Substrate kit (Vector Laboratories, SK-4100).

Primary antibodies used in this study were rabbit-anti-GABA (Sigma-Aldrich, A2052, 1:1,000), guinea pig-anti-GABA (Sigma-Aldrich, AB175, 1:1,000), mouse-anti-GAD67 (Millipore, MAB5406, 1:500), rabbit-anti-PV (Swant, PV27, 1:2,000), mouse-anti-PV (Millipore, P3088, 1:1,000), rabbit-anti-CCK (Immunostar, 20078, 1:200), mouse-anti-Calretinin (Millipore, MAB1568, 1:500), mouse-anti-Somatostatin (GeneTex, GTX1935, 1:500), rabbit-anti-Calbindin (abcam, ab11425, 1:1,000) rabbit-anti-GFP (Thermo Fisher, A11122, 1:1,000), chicken anti-GFP (Abcam, ab13970, 1:1,000), rabbit-anti-nNOS (Thermo Fisher, 61-7000, 1:500), mouse-anti-NeuN (Millipore, MAB377, 1:500), rabbit-anti-zsGreen (Takara, 632474, 1:500).

Secondary antibodies for immunofluorescence were from Jackson ImmunoResearch Labs and used at a concentration of 1:500: Alexa Fluor-488 donkey-anti-rabbit (705-545-003), Alexa Fluor-488 donkey-anti-guinea pig (706-545-148), Alexa Fluor-488 donkey-anti-mouse (715-545-150), Alexa Fluor-594 donkey-anti-mouse (715-585-150), and Alexa Fluor-647 donkey-anti-rabbit (711-605-152). Biotinylated goat anti-rabbit (BA-1000) and secondary antibodies for DAB staining were from Vector Laboratories and used at a concentration of 1:500.

#### RNAscope.

Fluorescent RNAscope in situ hybridization was performed according to the manufacturer’s instructions (Advanced Cell Diagnostics) with some modifications as described previously (25): in an RNase-free environment, 20-μm fixed brain sections were mounted on Superfrost Plus slides immediately after microtome sectioning and air-dried in a 60 °C oven for 30 min. Sections were rehydrated in PBS for 2 min and incubated for 5 min in 1X target retrieval solution at 95 °C. After one rinse in distilled water (2 min), sections were dehydrated with 100% ethanol for 5 s, air-dried, and incubated for 10 min in 5% hydrogen peroxide at 22 °C. Sections were then incubated in a HybEZ humidified oven at 40 °C with protease III for 30 min, and later with the probe solution for 2 h. After incubation with the probes, slides were incubated o/n in a solution of SSC5X at 22 °C. The next day, sections were incubated with the following solutions in a HybEZ humidified oven at 40 °C with three rinsing steps in between each: amplification Amp-1, 30 min; Amp-2, 30 min; Amp-3, 15 min. For each probe used, sections were incubated in a HybEZ humidified oven at 40 °C with the following solutions: HRP-C1, -C2, or -C3 (depending on the probe) for 15 min, Opal dye of choice for 30 min, and HRP-blocker for 15 min. Opal 520 (Akoya Biosciences, FP1487001KT), Opal 570 (Akoya Biosciences, FP1488001KT), and/or Opal 690 (Akoya Biosciences, FP1497001KT) dyes were used for fluorescent labeling. To achieve a fully amplified signal, the Opal dyes were used at a dilution of 1:1500.

The following probes were used: mouse Probe- Mm-Slc17a7 (VGLUT1) (Advanced Cell Diagnostics, 416631) and Probe- Mm-GAD1 (Advanced Cell Diagnostics, 400951).

#### TUNEL assay.

The in situ Cell Death Detection (TUNEL) Kit with TMR Red (Roche, 12156792910) was used to detect in situ apoptosis as described previously (21, 25). Briefly, 30 µm cryosections were refixed with 1% PFA for 20 min at 22 to 24 °C and rinsed with PBS (three times, 5 min each). Sections were then permeabilized in 0.1% sodium citrate and 1% Triton X-100 for 1 h at 22 to 24 °C. After rinsing in PBS (three times, 5 min each), sections were incubated with TUNEL reaction solution according to the vendor’s instruction, i.e., incubated in a mixture of 25 μL of terminal-deoxynucleotidyl transferase solution and 225 μL of label solution. Incubation was performed in a humidified chamber for 3 h at 37 °C in the dark. Sections were rinsed and mounted with Fluoromount containing DRAQ-5 (1:1,000). For a positive control, sections were treated with DNase I (10 U/mL, New England Biolabs, M0303S) for 1 h at 37 °C and rinsed in PBS (three times, 5 min each), followed by incubation with the TUNEL mixture.

### Imaging.

Images were acquired with a Leica SP5 confocal microscope with a 25×/0.95 water-immersion objective and a z resolution of 1 μm for immunohistochemistry and 0.7 μm for RNAscope, or with Leica Stellaris 5 with a 20×/0.75 CS2 dry objective and a z resolution of 1 μm for immunohistochemistry and 0.7 μm for RNAscope.

### Stereological Quantification.

All counts were performed by investigators double-blinded to the origin of each section using either Stereo Investigator or Image-J/Fiji.

Stereo Investigator software (MBF Bioscience) was used to exhaustively count DAB immunostained cells. Counting was carried out using optical fractionator sampling on a Zeiss Axioskop 2 microscope (40×/0.65 Ph2 objective) equipped with a motorized stage. The population of medial prefrontal cortex neurons was outlined on the basis of NeuN immunolabeling, with reference to a coronal atlas of the mouse brain and anatomical landmarks such as fiber tracts (79). To count GABAergic (GAD67+/PV+/CCK+) and glutamatergic neurons (VGLUT1+) in the sections, an adjacent section was stained for NeuN to define the ROI for the boundaries of mPFC. The area of the ROI and the cell density were compared between the control and experimental groups to ensure that the counted areas were comparable between the two groups and that changes in cell density were consistent with changes in the absolute counts. Pilot experiments with continuous counting determined that counting every 6th section was sufficient to estimate the number of GABAergic, glutamatergic neurons in the mPFC of each brain. Consequently, five to six sections were counted for each mouse brain. The average section thickness was measured prior to counting. Sections shrank from 30 μm to 20 to 22 μm after staining and dehydration. Exhaustive counting was performed, and no sampling grid was skipped. Counting was performed by two investigators double-blinded to the origin of the sections.

When using ImageJ/FIJI, fluorescent cell counts were performed by examining all sections within the confocal stacks without maximal projection as described earlier (25). Only neurons showing colocalization in at least three consecutive z-planes were included in the coexpression group. mPFC sections were analyzed from Bregma +2.8 mm to Bregma +1.54 mm, according to the Paxinos Mouse Brain Atlas. mPFC boundaries were determined based on the cytoarchitecture, as previously described (80, 81). 6-to-8 sections were counted for each mouse brain. The total number of coexpressing neurons was later calculated by multiplying the number of counted cells by 6-to-8. To check for equal sampling across experimental groups, the total number of mPFC ZsGreen+ neurons were counted in order to assure that their number was not significantly different across treatment groups.

#### Quantification of *Gad1* and *VglutT1* mRNA expression.

After RNAscope, seven optical sections (0.7 μm z step) of each physical section were examined as described previously (21, 25). Briefly, regions of interest (ROIs) were drawn to define the boundaries of mPFC using the Draq5 counterstain. Colocalization of ZsGreen+ cells with *GAD1*+ cells and with *VGluT1*+ cells was quantified using ImageJ with JACoP plugin. For cell counting, all slices within the confocal stack were examined without maximal projection. Images were analyzed exhaustively. This entailed scoring all neurons in every sixth section through the entire mPFC and reporting the number of cells for the number of mice examined. For each cell, boundaries were drawn using the optical section in which the cross-sectional area of the cell was the largest. The average area of ROIs for cells and the mPFC was consistent between the saline control and drug-treated groups. We included negative controls using a probe targeting a bacterial-origin gene *dapB* (Catalog number: 310043; GeneBank: EF191515) in each batch of experiments.

### Stereotaxic Injections and Viral Constructs.

Stereotaxic injections were performed as described previously (21, 25). Briefly, all surgical equipment was dry-heat sterilized using glass beads and the surgery area was disinfected with quaternary ammonium disinfectant (RX44). P7 to P8 pups were anesthetized using 3 to 4% vaporized Isoflurane and head-fixed on small animal stereotaxic apparatus (David Kopf Instruments Model 963) with nonrupture ear bars (Kopf model 922). Anesthesia was maintained throughout the procedure at a level that prevented reflex response to a tail/toe pinch, using a continuous flow of 1 to 2% vaporized Isoflurane. Eye drops were placed on each eye to prevent them from drying out, and vitals were checked every 10 min. The skin at the incision site was shaved with professional hair clippers. The bare skin was cleaned with ethanol and Betadine. Pups were placed on warming pads before and after injection. An incision was made to expose the skull from 2 to 3 mm anterior to bregma to 2 to 3 mm posterior to lambda. After removal of the fascia and cleaning with saline solution, 1 small burr hole (approx. 0.5 mm in diameter) was drilled in each hemisphere. Stereotaxic coordinates for the injection sites were determined using the Paxinos Brain Atlas and adjusted experimentally to x(M-L) = −0.183, y(R-C) = 1.335, z(D-V) = 0.6. A microsyringe (<0. 5 mm glass needle diameter, Warner Instruments, no. G150TF-4) was lowered through each of the holes, and a volume of 1 µL of a solution containing short-term HSV constructs (1 × 10^6^ infectious particles/mL) (HSV-DIO-GFP and HSV-DIO-GAD1-GFP were purchased from Viral Gene Transfer Core, Massachusetts Institute of Technology) were infused using a syringe pump (PHD Ultra™, Harvard apparatus, no. 70-3007) installed with a microliter syringe (Hampton, no.1482452A) at a rate of 100 nL/min. The syringe was left in place for 10 min to allow for diffusion. The needle was removed over 2 min, and the burr hole sealed with sterile bone wax. The skin incision was sutured with tissue adhesive glue (Vetbond tissue adhesive 1469SB). The incision site was treated with Betadine, and the animal was allowed to recover from anesthesia. At the end of the surgery, analgesic Buprenorphine-SR was administered to the animal (0.1 mg/kg subcutaneous injection) and the animal was placed on the heating pad for recovery.

### Statistics.

Statistical analyses of the data were performed using Prism 9 software and Excel Real Statistics package. The data were assessed and found to be in a normal distribution before appropriate parametric tests were applied. Details about the number of animals and statistical test used for each experiment are reported in the figure legends and Datasets S1 and S2. Means and SDs are reported for all experiments.

## Supplementary Material

Appendix 01 (PDF)

Dataset S01 (XLSX)

Dataset S02 (XLSX)

## Data Availability

All study data are included in the article and/or supporting information.
