# Supplementary material for: Embryonic exposure to environmental factors drives transmitter switching in the neonatal mouse cortex causing autistic-like adult behavior
Source: Proc Natl Acad Sci U S A. 2024 Aug 23;121(35):e2406928121. doi: 10.1073/pnas.2406928121 (PMC11363343; doi:10.1073/pnas.2406928121)
Supplement: Supplementary file 1 — Appendix 01 (PDF) [file pnas.2406928121.sapp.pdf]

PNAS

Supporting Information for

Embryonic exposure to environmental factors drives transmitter switching in neonatal mouse cortex causing autistic-like adult behavior

Swetha K. Godavarthi[1,2*], Hui-quan Li[1,2], Marta Pratelli[1,2], Nicholas C. Spitzer[1,2*]

[1]Neurobiology Department, University of California San Diego, La Jolla, CA 92093

[2]Kavli Institute for Brain & Mind, University of California San Diego, La Jolla, CA 92093

*Swetha K. Godavarthi- skgodavarthi@ucsd.edu and Nicholas C. Spitzer nspitzer@ucsd.edu

This PDF file includes:

Figures S1 to S9

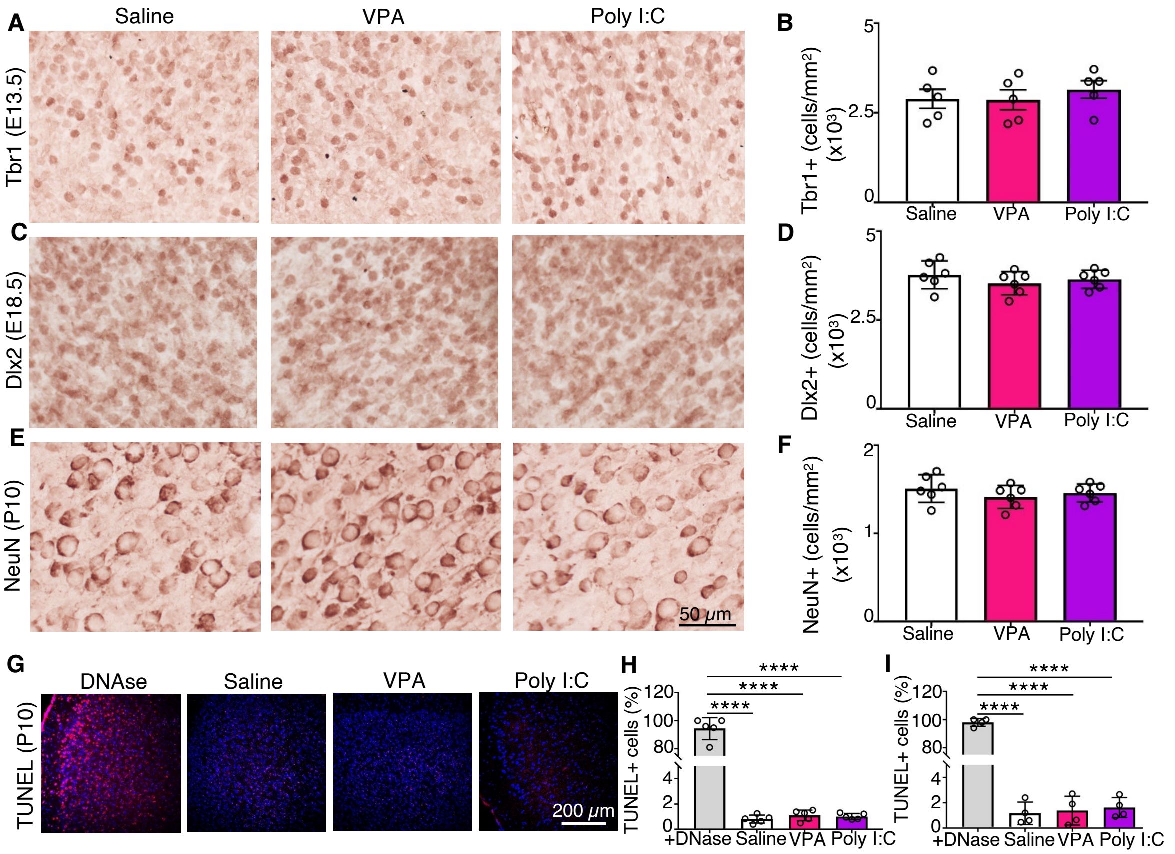

Fig S1. Prenatal exposure to VPA or Poly I:C does not alter the number of embryonic neuronal progenitors, or total neuron number or apoptosis in neonatal mice (**A, B**) Embryonic day (E) 13.5 pups from saline, VPA- and Poly I:C-treated groups were immunostained for Tbr1 in the telencephalon (A) and the number Tbr1+ cells/mm^2 was quantified (B). (**C, D**) E18.5 pups from saline, VPA- and Poly I:C-treated groups were immunostained for Dlx2 in the telencephalon (C) and the number Dlx2+ cells/mm^2 was quantified (D). (**E, F**) Postnatal day (P) 10 pups from saline, VPA- and Poly I:C-treated groups were processed for immunostaining for NeuN in the mPFC (E) and the number of NeuN+ cells/mm^2 was quantified (F). (**G**, **H**) Postnatal day (P) 10 pups from saline, VPA- and Poly I:C-treated groups were processed for TUNEL staining (magenta). (**I**) Quantification of TUNEL staining in P12 pups from saline, VPA- and Poly I:C-treated groups. n$\geq$5 mice. ****p<0.0001, one-way ANOVA followed by multiple comparison test (see Dataset S2 for details). Error bars: SD.

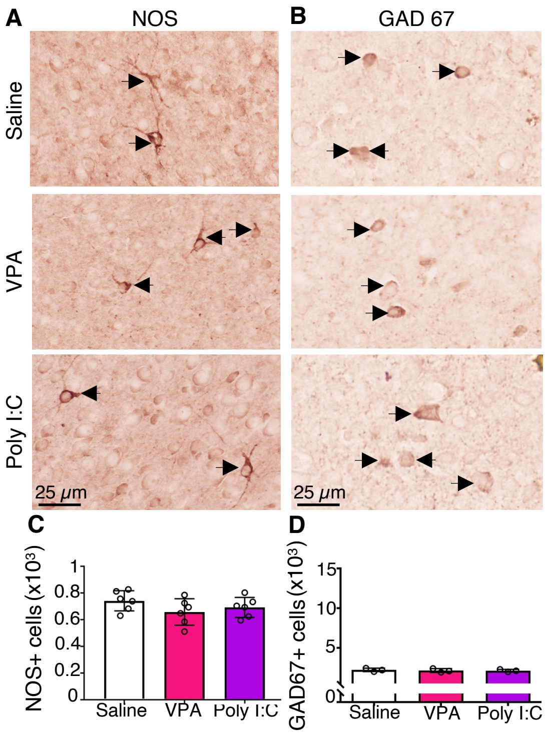

Fig S2. Prenatal exposure to VPA or Poly I:C does not alter the number of NOS1+ cells in the mPFC or GAD67+ cells in the lateral prefrontal cortex of neonatal mice (**A, B**) Postnatal day (P) 10 pups from saline, VPA- and Poly I:C-treated groups were processed for immunostaining for NOS1 in the mPFC (A) or GAD67 in the lateral PFC (B). Arrowheads indicate positive cells. (**C**, **D**) Stereological counts of NOS1+ and GAD67+ cells in mPFC and lateral PFC respectively of P10 pups. $n \geq 3$ mice. One-way ANOVA (see Dataset S2 for details). Error bars: SD.

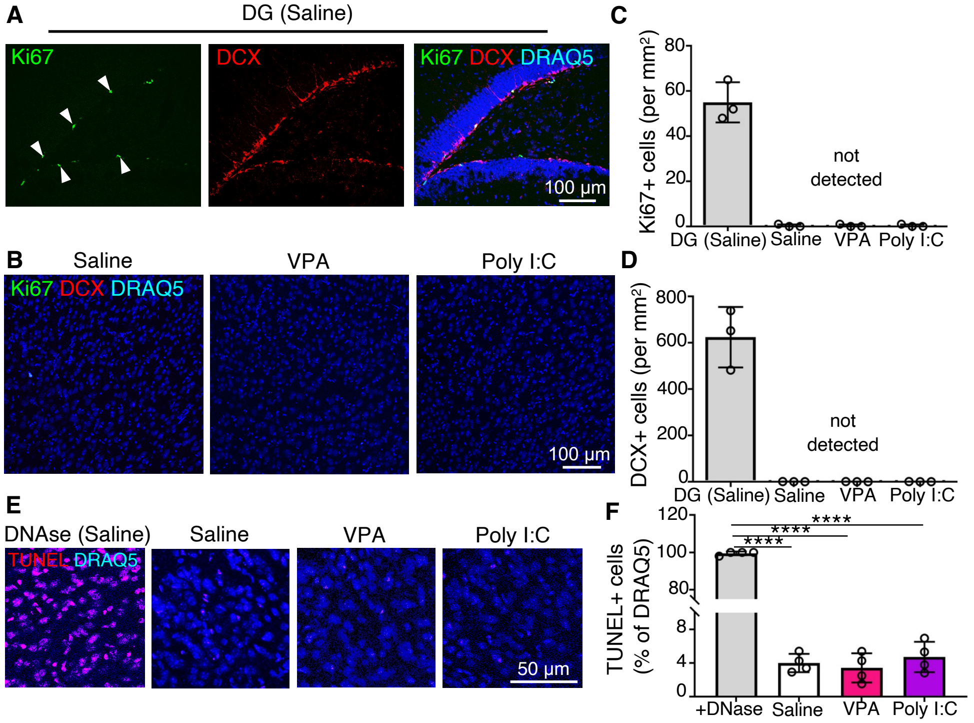

Fig S3. Prenatal exposure to VPA or Poly I:C does not alter the number of neuronal progenitors or apoptosis in P25 mice (**A**) Postnatal day (P) 25 pups from the saline-treated group were immunostained for Ki67 and DCX in the dentate gyrus (DG) as positive control. Arrowheads indicate Ki67+ cells (left panel). (**B**) P25 pups from saline, VPA- and Poly I:C-treated groups were immunostained for Ki67 and DCX in the mPFC. (**C, D**) Quantification of Ki67+ (C) and DCX+ (D) cells in the DG and mPFC. n=3 mice per group. (**E**, **F**) P25 pups from saline, VPA- and Poly I:C-treated groups were processed for TUNEL staining in the mPFC (E) and TUNEL signal quantified (F). ****p<0.0001, one-way ANOVA followed by multiple comparison (see Dataset S2 for details). n=4 mice per group. Error bars: SD.

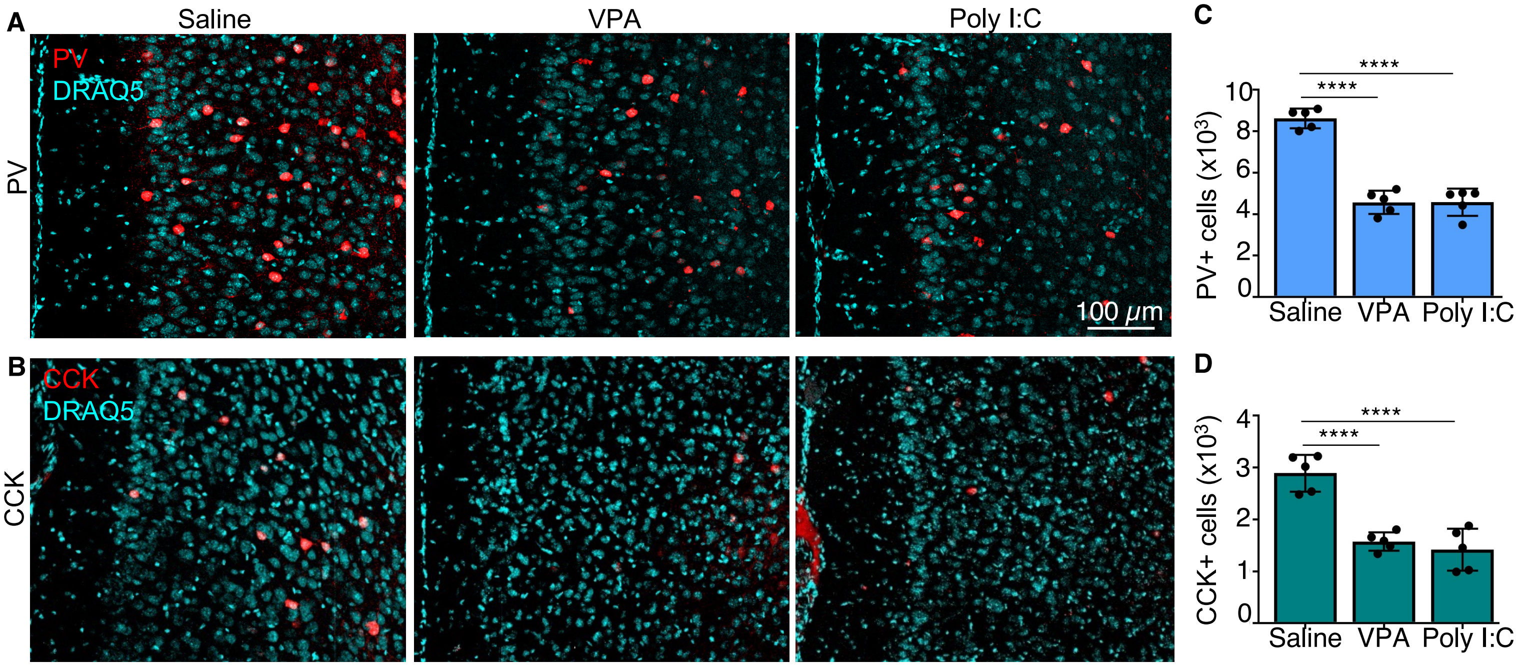

Fig S4. PV and CCK interneurons account for the decrease in number of GAD67+, GABA+ neurons in the mPFC of VPA- and Poly I:C-treated mice at P21. (**A-B**) Postnatal day (P) 21 C57BL6 mice from control, VPA and Poly I:C groups were immunostained for parvalbumin (PV, A) and cholecystokinin (CCK, B) in the mPFC. (**C-D**) Stereological counting of PV (C) and CCK (D) subtypes of GAD67 cells. n=5 for all. **** p<0.0001 with one-way ANOVA followed by multiple comparisons (see Dataset S2 for details). Error bar S.D.

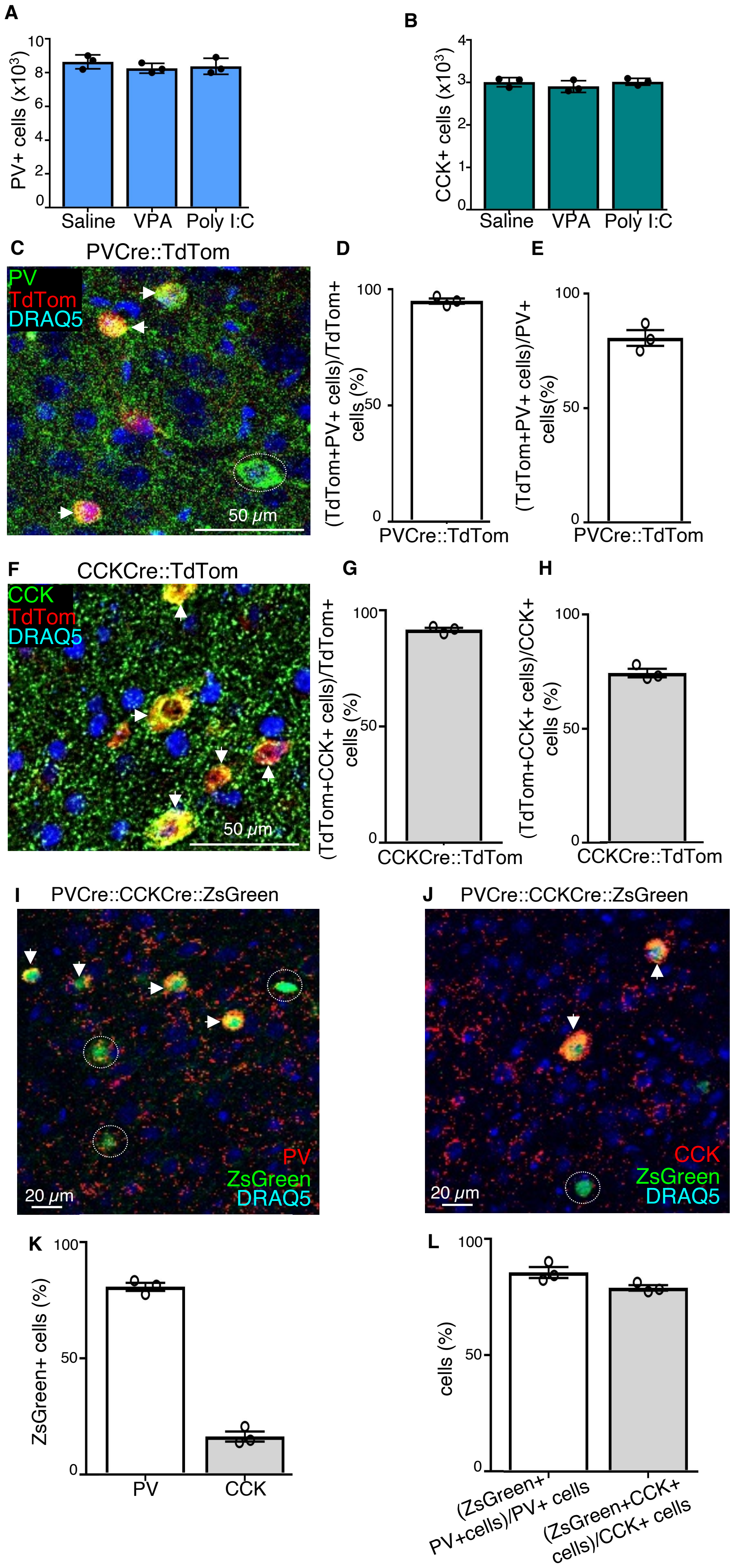
A
PV+ cells (x10³)
Saline
VPA
Poly I:C
B
CCK+ cells (x10³)
Saline
VPA
Poly I:C
C
PVCre::TdTom
PV
TdTom
DRAQ5
50 μm
D
(TdTom+PV+ cells)/TdTom+ cells (%)
PVCre::TdTom
E
(TdTom+PV+ cells)/PV+ cells(%)
PVCre::TdTom
F
CCKCre::TdTom
CCK
TdTom
DRAQ5
50 μm
G
(TdTom+CCK+ cells)/TdTom+ cells (%)
CCKCre::TdTom
H
(TdTom+CCK+ cells)/CCK+ cells (%)
CCKCre::TdTom
I
PVCre::CCKCre::ZsGreen
PV
ZsGreen
DRAQ5
20 μm
J
PVCre::CCKCre::ZsGreen
CCK
ZsGreen
DRAQ5
20 μm
K
ZsGreen+ cells (%)
PV
CCK
L
cells (%)
(ZsGreen+ PV+cells)/PV+ cells
(ZsGreen+CCK+ cells)/CCK+ cells

Fig S5. PV and CCK expression in drug-treated mice is not different from that in control mice at P90 and PVCre, CCKCre and PVCre::CCKCre mouse lines are specific and efficient for TdTomato expression at P21. (**A**) Stereological counting of PV cells in PVCre::TdTom Saline-, VPA- and Poly I:C-mice at postnatal day (P) 90. (**B**) Stereological counting of CCK cells in CCKCre::TdTom Saline-, VPA- and Poly I:C-mice at postnatal day (P) 90. (**C**) Postnatal day (P) 21 PVCre::TdTom pups were immunostained for parvalbumin (PV). Arrows indicate PV+TdTom+ cells, circles indicate PV+TdTom- cells. (**D**) Percentage of TdTom-labeled cells that are positive for PV (specificity). (**E**) Percentage of PV-labelled cells that are positive for TdTom (efficacy). (**F**) Postnatal day (P) 21 CCKCre::TdTom pups were immunostained for cholecystokinin (CCK). Arrows indicate CCK+TdTom+ cells. (**G**) Percentage of TdTom-labeled cells that are positive for CCK (specificity). (**H**) Percentage of CCK-labelled cells that are positive for TdTom (efficacy). (**I**) Postnatal day (P) 21 PVCre::CCKCre::ZsGreen pups were immunostained for PV. Arrows indicate PV+ZsGreen+ cells, circles indicate PV-ZsGreen+ cells. (**J**) Postnatal day (P) 21 PVCre::CCKCre::ZsGreen pups were immunostained for CCK. Arrows indicate CCK+ZsGreen+ cells, circles indicate CCK-ZsGreen+ cells. (**K**) Percentage of ZsGreen-labeled cells that are positive for PV or CCK (specificity). (**L**) Percentage of either PV or CCK-labelled cells that are positive for ZsGreen (efficacy). Nuclei of all sections are counterstained for DRAQ5. One-way ANOVA followed by multiple comparisons. Error bar S.D. n=3 for all groups (see Dataset S2 for details).

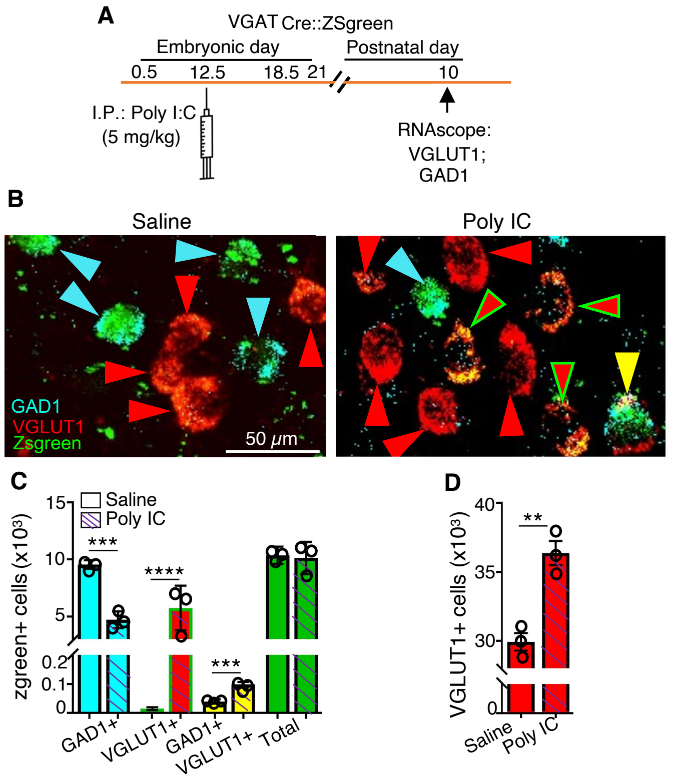

Fig S6. Gain of VGLUT1 occurs in the same VGAT-Cre::ZSGreen neurons losing GAD1. (**A**) Experimental protocol. Following a single intraperitoneal (IP) dose of Poly I:C in pregnant dams at embryonic day (E) 12.5, VGATCre-Zsgreen transgenic pups were perfused at postnatal day (P) 10 for RNAscope for VGLUT1 and GAD1. (**B**) Zsgreen+GAD1+ cells (cyan arrowheads) along with VGLUT1+ only cells (red arrowheads) are observed in saline-exposed (control) pups. Zsgreen+GAD1-VGLUT1+ cells (red arrowheads with green border) and Zsgreen+GAD1+VGLUT1+ cells (yellow arrowheads) are additionally detected in Poly I:C-treated pups. (**C**) Stereological counts of subtypes of Zsgreen+ cells: GAD1+, VGLUT1+, and GAD1+VGLUT1+. n=3. ***p<0.001, **** p<0.0001, two-way ANOVA followed by multiple comparison (see Dataset S2 for details). (**D**) total VGLUT1+ cells. n=3. **p<0.01, unpaired two-tailed t-test. Error bars: SD.

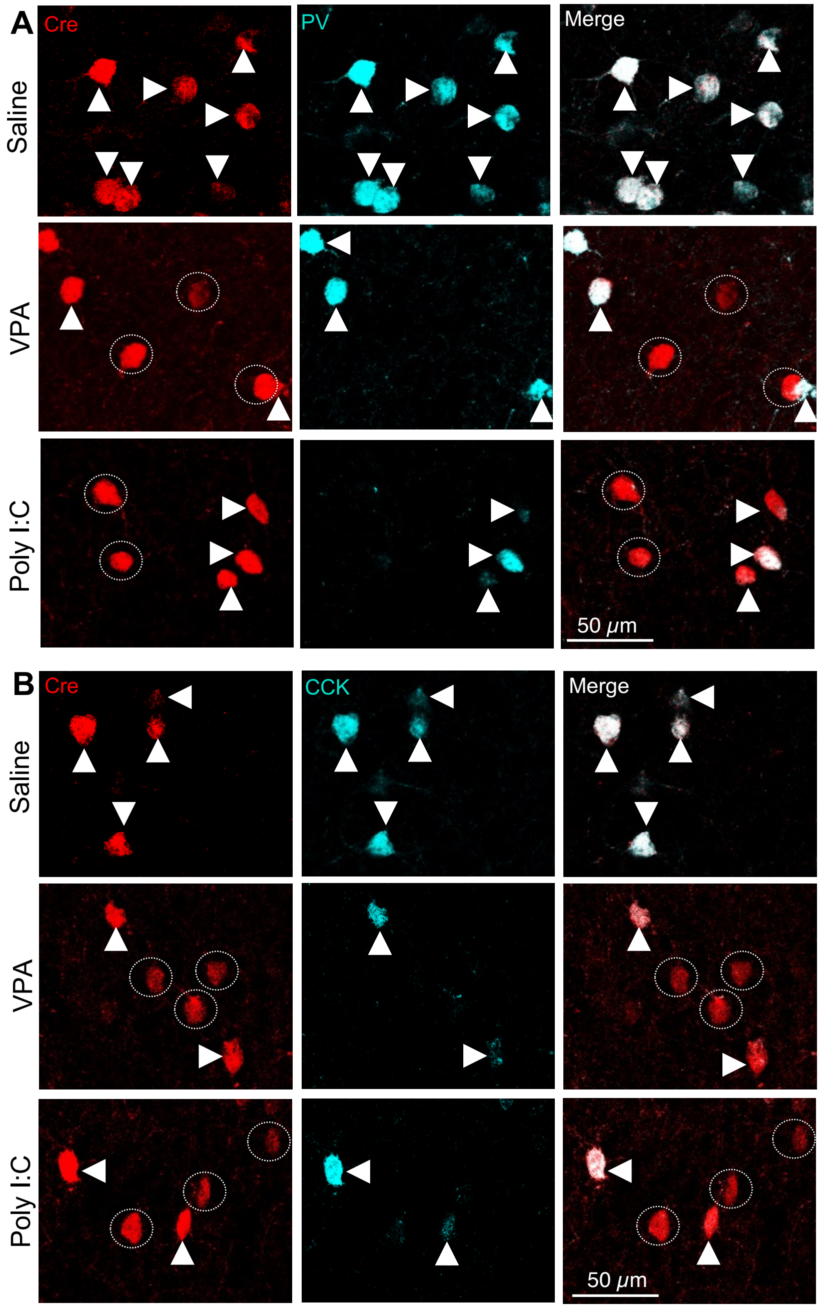

Fig S7. Persistent Cre recombinase expression with reduced detection of PV or CCK in PVCre and CCKCre mice following treatment with VPA or Poly I:C. (**A**) Saline- VPA- or Poly I:C-treated postnatal day (P) 10 PV-Cre pups were immunostained for parvalbumin (PV) and Cre recombinase (Cre). Arrowheads indicate PV+Cre+ cells and circles indicate PV-Cre+ cells in VPA- and Poly I:C-treated mice. n=3. (**B**) Saline-, VPA- or Poly I:C-treated P10 CCK-Cre pups were immunostained for cholecystokinin (CCK) and Cre recombinase (Cre). Arrowheads indicate CCK+Cre+ cells and circles indicate CCK-Cre+ cells in VPA- and Poly I:C-treated mice. n=3. PVCre::CCKCre mice were not tested for Cre recombinase expression, as the saline group is expected to yield PV-Cre+ cells (from CCK+ cells) and CCK-Cre+ cells (from PV+ cells), hindering assessment of maintenance of Cre recombinase activity in the cell types with PV or CCK expression below the detection limit.

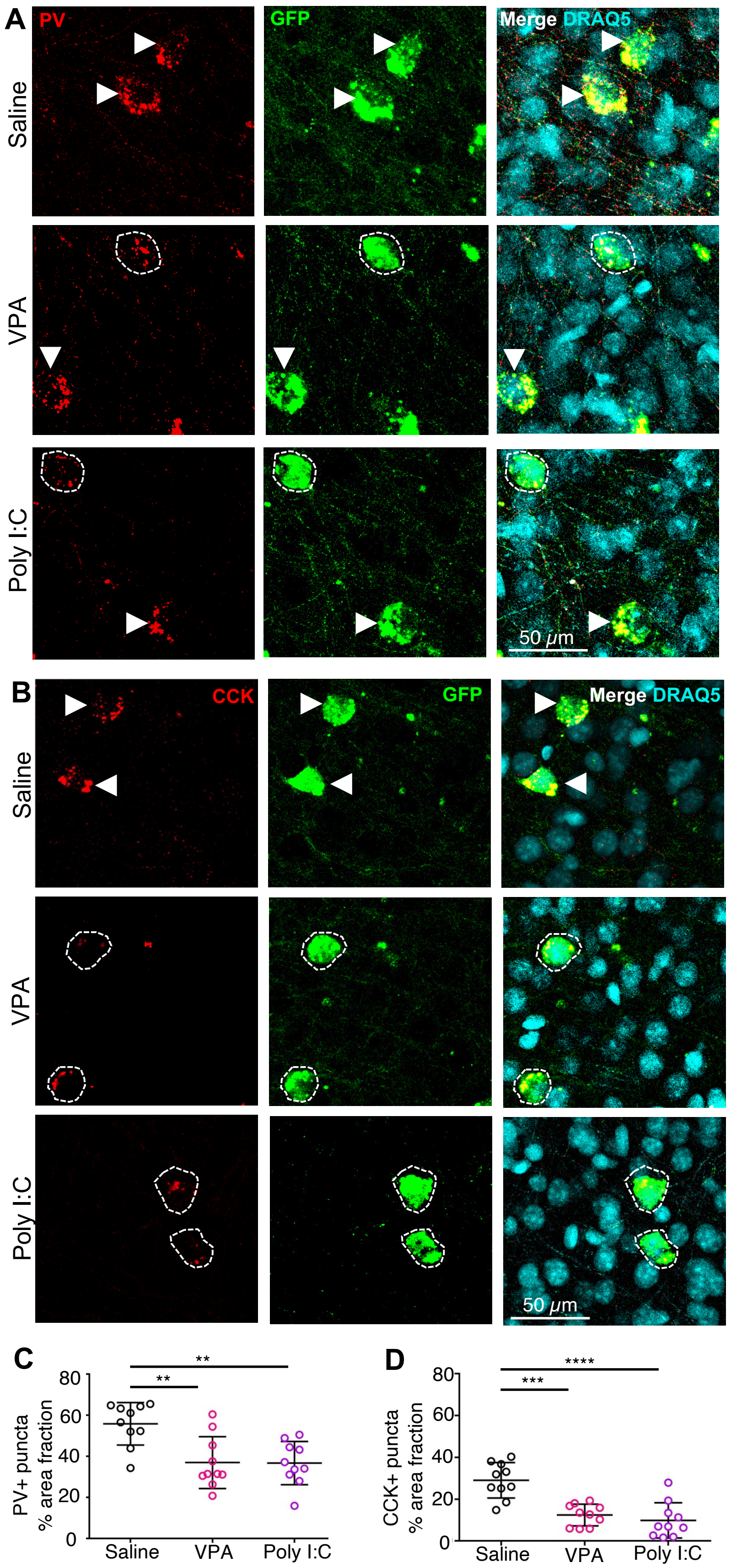

Fig S8. Persistent Cre recombinase reporter (GFP) expression with reduced detection of PV or CCK transcripts in PVCre::ZsGreen and CCKCre::ZsGreen mouse lines following treatment with VPA or Poly I:C. (**A**) Saline-, VPA- or Poly I:C-treated postnatal day (P) 10 PV-Cre pups were probed for parvalbumin (PV) puncta with RNAscope, immunostained for GFP and counterstained with DRAQ5. Arrowheads indicate PV+GFP+ cells and circles indicate GFP+ cells in VPA- and Poly I:C-treated mice with fewer PV puncta. (**B**) Saline-, VPA- or Poly I:C-treated P10 CCK-Cre pups were probed for cholecystokinin (CCK) puncta with RNAscope, immunostained for GFP and counterstained with DRAQ5. Arrowheads indicate CCK+Cre+ cells and circles indicate GFP+ cells in VPA- and Poly I:C-treated mice with fewer CKK puncta. (**C,D**) Quantification of PV (C) and CCK (D) expression measured as percent of cell area occupied by mRNA puncta across mPFC GFP+ cells. n≥3 cells per mouse for 3 mice. $^{**}p<0.01$, $^{***}p<0.001$, $^{****}p<0.0001$, one-way ANOVA followed by multiple comparisons test (see Dataset S2 for details). Error bars: SD.

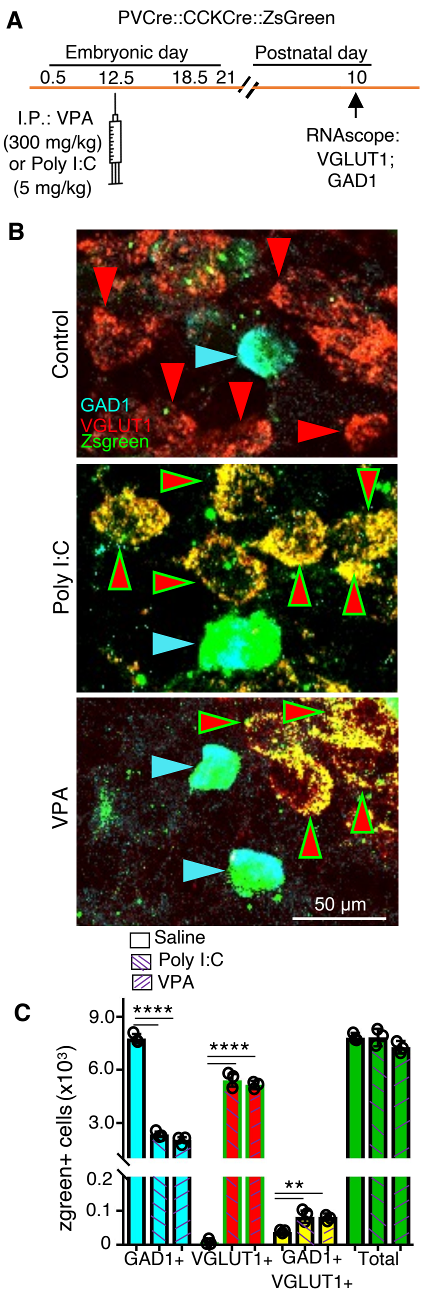

Fig S9. Gain of VGLUT1 occurs in the same PVCre::CCKCre::ZsGreen mPFC neurons that lose GAD1. (**A**) Experimental protocol. Following a single intraperitoneal (IP) dose of VPA or Poly I:C in pregnant dams at embryonic day (E) 12.5, PVCre::CCKCre::Zsgreen transgenic pups were perfused at postnatal day (P) 10 for RNAscope for VGLUT1 and GAD1. (**B**) Zsgreen+GAD1+ cells (cyan arrowheads) along with VGLUT1+ only cells (red arrowheads) are observed in control pups. Zsgreen+ GAD1- VGLUT1+ cells (red arrowheads with green border) are additionally detected in Poly I:C-treated pups. (**C**) Stereological counts of subtypes of Zsgreen+ cells: GAD1+, VGLUT1+, and GAD1+VGLUT1+. n$\geq$3. **p<0.01, **** p<0.0001, two-way ANOVA followed by multiple comparison (see Dataset S2 for details). Error bars: SD.
